# Lipo-chitooligosaccharide and thuricin 17 act as plant growth promoters and alleviate drought stress in *Arabidopsis thaliana*

**DOI:** 10.3389/fmicb.2023.1184158

**Published:** 2023-08-04

**Authors:** Sowmyalakshmi Subramanian, Erika Mitkus, Alfred Souleimanov, Donald L. Smith

**Affiliations:** ^1^Department of Plant Sciences, MacDonald Campus, McGill University, Montreal, QC, Canada; ^2^Department of Biology, McGill University, Montreal, QC, Canada

**Keywords:** *Arabidopsis thaliana*, lipo-chitooligosaccharide, thuricin 17, phytohormones, drought stress, mass spectrometry

## Abstract

Lipo-chito-oligosaccharide (LCO–from *Bradyrhizobium japonicum*) and thuricin 17 (Th17–from *Bacillus thuringiensis*) are bacterial signal compounds from the rhizosphere of soybean that have been shown to enhance plant growth in a range of legumes and non-legumes. In this study, an attempt to quantify phytohormones involved in the initial hours after exposure of *Arabidopsis thaliana* to these compounds was conducted using UPLC-ESI-MS/MS. A petri-plate assay was conducted to screen for drought stress tolerance to PEG 8000 infusion and plant growth was studied 21-days post-stress. *Arabidopsis thaliana* plants grown in trays with drought stress imposed by water withhold were used for free proline determination, elemental analysis, and untargeted proteomics using LC-MS/MS studies. At 24 h post-exposure to the signal compounds under optimal growth conditions, *Arabidopsis thaliana* rosettes varied in their responses to the two signals. While LCO-treated rosettes showed a decrease in total IAA, cytokinins, gibberellins, and jasmonic acid, increases in ABA and SA was very clear. Th17-treated rosettes, on the other hand, showed an increase in IAA and SA. Both treatments resulted in decreased JA levels. Under severe drought stress imposed by PEG 8000 infusion, LCO and Th17 treatments were found to significantly increase fresh and dry weight over drought-stressed control plates, indicating that the presence of the signaling compounds decreased the negative effects experienced by the plants. Free proline content increased in LCO- and Th17-treated plants after water-withhold drought stress. Elemental analysis showed a significant increase in carbon percentage at the lower concentration of Th17. Untargeted proteomics revealed changes in the levels of drought-specific ribosomal proteins, glutathione S-transferase, late embryogenesis proteins, vegetative storage proteins 1 and 2, thaumatin-like proteins, and those related to chloroplast and carbon metabolism. The roles of some of these significantly affected proteins detected under drought stress are discussed.

## 1. Introduction

Population growth and urbanization, water pollution, and shrinking irrigated land are all occurring at an alarmingly rapid rate, and more than 60% of the world's land is negatively impacted by water availability (Meena et al., 2017). In the years between 2000 and 2019, value-added agriculture has increased by 73%, the use of pesticides by 36%, and nitrogen fertilizer use by 57%, all contributing to a 53% increase in primary crop production (FAO, 2021). Because of this, the global demand for water for agriculture currently amounts to 70% (Boretti and Rosa, 2019; Kopecká et al., 2023). Water use from aquifers to yield to the increasing demand for more food production and the climate change patterns have affected agriculture globally, and Sub-Saharan Africa will be especially vulnerable to the negative effects of climate change (Huntingford et al., 2005; Orimoloye, 2022). Multi-scale drought analysis suggests patterns of multi-crop susceptibility to drought, with wheat, soybean, and corn crop yields being the most affected (Hendrawan et al., 2022; Santini et al., 2022). Global climate models (GCMs) indicate that global croplands and pasture lands will be affected by climate change due to water scarcity and a decrease in total agriculture area between 2010 and 2050 (Fitton et al., 2019; Rojas, 2020). FAO statistics confirm the projection that despite the increase in food production, the agriculture cropland has indeed reduced by 3% since 2000 (FAO, 2021).

Climate change effects are greater at higher latitudes and will affect the polar regions of the North profoundly (Beck et al., 2018). It is estimated that Canada will experience an increase in temperature of 5–8°C over the course of this century. During the last century, the southern regions of Canada, which include areas of intense agriculture, have seen increases of 0.5–1.5°C in mean annual temperature. This has often been accompanied by varying annual precipitation increases, in the range of 5–35%, with the greatest increases seen during the winter (Almaraz et al., 2008). However, drought situations in 2001–2002, followed by the recent 2021–2022 water-stressed agriculture conditions in Canada, reflect how profound the changes in climate can be for Canadian agriculture. Better management practices, based on crop sensitivity to temperature and water availability, and the use of sustainable biologicals can significantly reduce crop loss (Gornall et al., 2010; Ilangumaran and Smith, 2017).

One of the central themes of plant growth and development is the cognition of intracellular regulatory mechanisms, of which phytohormones form an important component. During plant–microbe co-evolution, plants, and the associated microbes have each expanded their receptors and response systems to form a complex network of signaling, contributing to the fitness of the interaction partners. There is always a concerted effort to grow and develop while constantly monitoring the various environmental factors, both useful and harmful. Phytohormones coordinate with these signaling events and act according to the cues received (Nobori et al., 2018; Figueroa-Macías et al., 2021). Ever since salicylic acid (SA) was reported to induce systemic acquired resistance (SAR) in tobacco (White, 1979), both SA (Conrath et al., 1995; Wildermuth et al., 2001) and jasmonic acid (Wasternack and Hause, 2013; Wasternack and Strnad, 2018; Gomi, 2021) have been shown to play significant roles both in plant growth promotion and their levels are elevated for phyto-protection during pathogen interactions. These interactions are closely associated with levels of other phytohormones such as ethylene (ETH), abscisic acid (ABA), and auxin (Nobori et al., 2018). Cross talk between cytokinins and SA has been found to modulate many biotrophic pathogens (Albrecht and Argueso, 2017 and the references therein). For example, pathogens, such as *Pseudomonas syringae* DC 3000, mimic phytohormones to dampen plant immunity for efficient infection (Weiler et al., 1994; Mittal and Davis, 1995; Albrecht and Argueso, 2017; Huot et al., 2017).

Not only do the hormones engage in extensive cross talk but also regulate plant growth by integrating these signals. These low molecular weight chemicals can modify basic physiological functions in varying amounts at different stages of the plant's life cycle. Apart from the five basic hormones [auxin (indole acetic acid—IAA), cytokinins (Cyt), gibberellins (GA), abscisic acid (ABA), and ethylene (ETH)], brassinosteroids, salicylic acid (SA), jasmonic acid (JA), strigolactones, and fusicoccins have all added to the complexity of the phytohormones network and signaling. Phytohormones maintain the circadian rhythm by adjusting the clocks to environmental cues (Robertson et al., 2009). While auxins regulate the amplitude and precision of the circadian clock, brassinosteroids and ABA regulate circadian periodicity and cytokinins delay the circadian cycle using ARABIDOPSIS RESPONSE REGULATOR 4 and the photoreceptor phytochrome B (Hanano et al., 2006; Seung et al., 2012). A large variety of developmental processes and adaptive responses to external environmental cues are also controlled by ABA. ABA in *Arabidopsis thaliana* affects at least 10% of the protein-coding genes, making it by far the largest percentage of gene regulation among phytohormones. Transcription factors, such as bZIP and AREB/ABFs, are controlled in an ABA-responsive-element (ABRE) dependent manner during seed germination and in the vegetative stages during osmotic stress, while other transcription factors, such as AP2/ERF, MYB, NAC, and HD-ZF, participate in ABA regulation based on circadian rhythm and light perception (Fujita et al., 2011). During abiotic stress responses, thiamine compounds (vitamin B1) and thiamine di-phosphate dependent enzymes are synthesized to overcome some of the oxidative stress effects and are controlled by ABA (Rapala-Kozik et al., 2012). Cross talk between ABA and GA in endodermal cells of the roots restricts root growth under salinity stress. The quiescence is controlled by ABA which represses root growth (Duan et al., 2013). When faced with dehydration, either ABA-dependent or ABA-independent pathways may be activated, each of which transcriptionally controls specific drought stress-related genes in *Arabidopsis* (Yamaguchi-Shinozaki and Shinozaki, 1994, 2007; Shinozaki and Yamaguchi-Shinozaki, 1997). An important factor in activating the dehydration stress pathway is the gene DREB2, a trans-acting factor that binds to the dehydration response element (DRE) and induces a signal transduction pathway (Liu et al., 1998). These gene pathways can produce a wide variety of products, including osmolytes such as proline, sugars such as trehalose, antioxidants, water channel proteins, potassium transporters, proteases, and ACC deaminase (Matsui et al., 2008; Orozco-Mosqueda et al., 2022 and references therein). PEG (polyethylene glycol) 8000 osmotically lowers the water potential of the growth medium and thus induces drought stress in the plants. A study on the effect of PEG on light-grown *Arabidopsis* plants produced plants that showed primary root elongation and smaller root diameter than controls, when under a moderate water deficit. The plants growing under a moderate water deficit had enhanced rates of cell production (van der Weele et al., 2000).

With recent advances in instrumentation and detection techniques, it is now possible to understand some of the plant–microbe interactions in a more detailed manner. Plant growth-promoting rhizobacteria (PGPR) employ a variety of mechanisms to promote plant growth and development such as enhanced nutrient availability (inclusive of nitrogen fixation), suppression of diseases through a range of biocontrol mechanisms, induction of disease resistance in plants, production of phytohormones, and production of signal compounds, including volatile forms. As bio-fertilizers (made with living microorganisms), some PGPR promote plant growth and make available essential nutrients from the environment that are otherwise unavailable to plants due to their conjugated or precipitated forms (Glick, 2012; Backer et al., 2018; Lyu et al., 2020, 2021; Naamala and Smith, 2020; Shah et al., 2021).

Currently, two compounds secreted by the rhizosphere bacteria of soybean, namely lipo-chito-oligosaccharide (LCO) from *Bradyrhizobium japonicum* 532C, Nod Bj V (C18:1; MeFuc) (Prithiviraj et al., 2000), and thuricin 17 (Th17) from *Bacillus thuringiensis* NEB17 (Gray et al., 2006a,b), are under evaluation to allow unraveling the mechanism of action in plant growth promotion. *Bradyrhizobium japonicum* 532C is a Hup^−^ strain, also known as 61A152, that has adapted well to Canadian soils. Multiple field trial evaluations in the early 1980s in sites close to Guelph, Ontario, for soybean yields, suggested this strain to be superior due to its consistent performance across years and locations (Hume and Shelp, 1990). It has since been used as a single-strain inoculum commercially. *Bradyrhizobium japonicum* 532C was procured from Nitragin Company Inc., Milwaukee, Wisconsin, USA; the culture is maintained regularly in our laboratory to isolate LCO.

Lipo-chito-oligosaccharide are oligosaccharides of β-1,4-linked N-acetyl-D-glucosamine coded for by a series of *nod* genes and are rhizobia-specific. These are regulated by plant-rhizobia-specific signals such as isoflavonoids (Vazquez et al., 1993; Carlson et al., 1994; Spaink et al., 1995; Schultze and Kondorosi, 1996, 1998; Kamst et al., 1998). Nod Bj V (C18:1; MeFuc) contains a methyl-fucose group at the reducing end that is encoded by the host-specific *nodZ* gene (López-Lara et al., 1995), which is an essential component of soybean–rhizobia interactions. Apart from being nodulation signals, LCOs positively affect plant growth and development in non-legumes. The potential role of LCOs in plant growth regulation was first reported by Denarie and Cullimore (1993). These effects can be produced even with extremely low concentrations of purified LCOs, as low as 10^−12^ M (Hungria and Stacey, 1997). Plant defense-related responses, such as chitinase and PR proteins (Schultze and Kondorosi, 1996, 1998) and peroxidase (Cook et al., 1995), and enzymes of the phenylpropanoid pathway, such as L-phenylalanine ammonia-lyase (PAL) (Inui et al., 1997; Wang et al., 2012), have been reported. Under optimal conditions, LCOs induced root growth (Souleimanov et al., 2002a,b), increased photosynthetic rate in greenhouse-grown corn (Khan, 2003), and increased flowering and yield of tomato (Chen et al., 2007). Seed germination and seedling establishment under optimal and abiotic stress conditions were enhanced in common bean, corn, rice, apple, grapes (Zhang and Smith, 2001; Atti et al., 2005; Khan et al., 2010), soybean (Prudent et al., 2014; Subramanian et al., 2016a), canola (Schwinghamer et al., 2015, 2016), *Arabidopsis* (Subramanian et al., 2016b), and switchgrass (Arunachalam et al., 2018). This volume of work has gone far to prove that purified LCOs can have significant physiological and developmental effects on non-legume plants.

*Bacillus thuringiensis* NEB17 was isolated from soybean root nodules as putative endophytic bacteria in 1998 in our laboratory and was deposited at the International Depositary Authority of Canada (IDAC) in March 2003, with the Accession no. 270303-02. When co-inoculated with *B. japonicum* under nitrogen-free conditions, the bacteria promoted soybean growth, nodulation, and grain yield (Bai et al., 2002, 2003). The causative agent of plant growth promotion is a bacteriocin thuricin 17 (Th17) (Gray et al., 2006b), which has been shown to exhibit functional similarities and anti-microbial activities with bacthuricin F4 produced by *B. thuringiensis* subsp. kurstaki BUPM4 (Jung et al., 2008). Genes encoding the bacteriocin were deposited at the National Center for Biotechnology Information (NCBI) in May 2009 as FJ159242. Thuricin 17, when applied as leaf spray and root drench, had positive effects on soybean and corn growth (Lee et al., 2009), the first report of plant growth stimulation by a bacteriocin. Plant growth promotion and abiotic stress alleviation studies comparing LCO and Th17 responses have been conducted on *Arabidopsis thaliana* (Subramanian et al., 2016b), soybean (Prudent et al., 2014; Subramanian et al., 2016a), and canola (Schwinghamer et al., 2015, 2016; Nazari et al., 2022) under controlled conditions and on soybean, potato (Gautam et al., 2016), and switchgrass in the field (Arunachalam et al., 2018).

In this study, the aim was to understand the effects of the bacterial signal compounds LCO and Th17 on the plant model system *Arabidopsis thaliana*. We hypothesized that LCO and Th17 will differ in conferring phytohormone responses in optimally grown *Arabidopsis thaliana* rosettes. If the responses were found to be different, the pattern of abiotic stress response via untargeted proteomics would suggest how the biostimulants help plants respond to drought stress.

## 2. Materials and methods

### 2.1. Plant growth conditions

Seeds of *Arabidopsis thaliana* Col-0 were procured from Lehle Seeds (Round Rock, TX, USA), and plants were then propagated in a growth chamber to produce more seeds. The seeds of *Arabidopsis thaliana* Col-0 were planted in peat pellets and grown in a growth chamber at 22 ± 2°C with a photoperiod of 16/8-h day/night cycle and under 100–120 μmol quanta m^−1^ s^−1^, at 65–70% relative humidity. Three-week-old plants were used for the UPLC-ESI/MS hormone analyses, drought stress, and label-free proteomics. Treatment administration and sampling time for all the experiments were always conducted between 08:00 and 08:30 h to be consistent with the application within the circadian rhythm cycle of the plants.

### 2.2. Extraction and purification of lipo-chitooligosaccharides and thuricin 17

The extraction and purification of LCOs from *Bradyrhizobium japonicum* strain 532C followed the method of Souleimanov et al. (2002b). The identification of Nod factors was conducted by comparing the retention time of isolated Nod factors with standard Nod factors, also from strain 532C, and identified by mass spectrometry. *Bacillus thuringiensis* NEB17 was cultured in King's medium (King et al., 1954) as previously described (Gray et al., 2006a). Th17 isolation and purification were carried out using high-performance liquid chromatography (HPLC) following the procedures of Gray et al. (2006b). The collected material was denoted by partially purified Th17 and stored at 4°C and diluted to the required concentrations for all experiments.

In the experiments, LCO concentrations of 10^−6^ M (LCOA) and 10^−8^ M (LCOB) and Th17 concentrations of 10^−9^ M (THA) and 10^−11^ M (THB) were used. Among these, LCO 10^−6^ M and Th17 10^−9^ M were previously found to be the best for plant growth response studies (Souleimanov et al., 2002a; Prithiviraj et al., 2003; Atti et al., 2005; Lee et al., 2009; Subramanian et al., 2016a,b).

### 2.3. Phytohormone quantification

#### 2.3.1. Sampling of *Arabidopsis thaliana* rosettes for hormone analysis

For the UPLC-ESI/MS-MS analysis, *Arabidopsis thaliana* plants were grown in large trays. Fifteen-day-old plants were transferred to trays to group 10 plants each per technical replicate. Three such trays per treatment were organized in the growth chamber at random, per experiment replicate. Three-week-old plants were treated with LCOA (10^−6^ M) and THA (10^−9^ M) as root drench (@ 15 ml per plant) with water being the control treatment. Twenty-four hours after the treatment, *Arabidopsis thaliana* rosettes were sampled (each of the treatment replicates was pooled to get 30 plants) and lyophilized to obtain a homogenous sample. Approximately 1 g of the lyophilized material was used for quantification (Savant Modulyo, Model VLP285 Valu pump, Savant Instruments Inc., NY, USA). Fresh rosettes were sampled and flash-frozen in liquid nitrogen for SA and JA analyses. Five biological replicates were analyzed by this method.

#### 2.3.2. Extraction and purification

In brief, a 100 μl aliquot containing all the internal standards, each at a concentration of 0.2 pg μl^−1^, was added to approximately 50 mg of homogenized plant tissue; 3 ml of isopropanol:water:glacial acetic acid (80:19:1, v/v) was then added, and the sample was agitated in the dark for 24 h at 4°C. Samples were centrifuged, and the supernatant was isolated and dried on a Büchi Syncore Polyvap (Büchi, Switzerland). Samples were reconstituted in 100 μl acidified methanol (10% glacial acetic acid in Methanol v/v), adjusted to 1 ml with acidified water (5% glacial acetic acid in water v/v), and then partitioned against 2 ml hexane. After 30 min, the aqueous layer was isolated and dried as above. Dry samples were reconstituted in 800 μl acidified methanol and adjusted to 1 ml with acidified water. The reconstituted samples were passed through equilibrated Sep-Pak C18 cartridges (Waters, Mississauga, ON, Canada), the eluate being dried on a LABCONCO centrivap concentrator (Labconco Corporation, Kansas City, MO, USA). An internal standard (blank) was prepared with 100 μl of the deuterated internal standard mixture (Abrams et al., 2003; Zaharia et al., 2005). A QC (quality control) standard was prepared by adding 100 μl of a mixture containing all the analytes of interest, each at a concentration of 0.2 pg μl^−1^, to 100 μl of the internal standard mix. Finally, samples, blanks, and QCs were reconstituted in a solution of 40% methanol (v/v), containing 0.5% acetic acid and 0.1 pg μl^−1^ of each of the standards.

For the SA/JA analysis, the plant material was ground to a fine powder in liquid nitrogen with a mortar and pestle. Frozen plant material (~500 mg) was extracted with a mixture of methanol:water:glacial acetic acid (3 ml, 90:9:1, v/v/v), to which the internal standards were added (100 L solution acetonitrile:water, 50:50 v/v, with 0.1% formic acid, containing 1 ng L^−1^ of 3,4,5,6-*d*4-2-hydroxybenzoic acid and 0.5 ng L^−1^ of 2,2-*d*2- jasmonic acid). Following sonication (5 min) and incubation on an orbital shaker (4°C, 5 min), the samples were centrifuged (4.4 k rpm, 10 min) to pellet the debris. The supernatant was transferred to a clean tube, the pellets were re-suspended in the extraction solution (2 ml), and the procedure was repeated. The supernatant was combined with the initial extracted volume, and the pellet was re-suspended in methanol (1 ml). The extraction step was repeated a third time. After the supernatants were combined, methanol was evaporated under reduced pressure. On the ice, aqueous NaOH (1 ml, 0.3 N) was added to each sample, which was further extracted with dichloromethane (3 ml). The aqueous layer was transferred to a clean tub, while the organic layer was re-extracted with aqueous NaOH (2 ml). On the ice, combined aqueous layers were acidified with 5% aqueous HCl (1 ml); then, they were extracted with a mixture of ethyl acetate:cyclohexane (1 ml, 1:1, v/v). The organic phase was collected, and the aqueous phase was extracted a second time with the same mixture (0.5 ml). The organic fractions were pooled, and the solvent was evaporated under a constant nitrogen stream. Before mass spectrometric analysis, the samples were reconstituted in a mixture of methanol:water (200 L, 30:70, v/v) containing 0.1% formic acid, to which external standards were added (100 ng of 1,2,3,4,5,6-13*C*6-2-hydroxybenzoic acid and 50 ng of 12,12,12-*d*3-jasmonic acid) (Galka et al., 2005).

#### 2.3.3. Hormone quantification by UPLC-ESI-MS/MS

A detailed description of the procedure for quantification of multiple hormones and metabolites, including auxins (IAA, IAA-Asp, and IAA-Glu), abscisic acid and metabolites (ABA, PA, DPA, 7′-OH-ABA, neo-PA, and ABA-GE), cytokinins (2iP, iPA, Z, ZR, dhZ, dhZR, and Z-O-Glu), and gibberellins (GAs 1, 3, 4, 7), is available in Chiwocha et al. (2003, 2005) study. Samples were injected onto an ACQUITY UPLC^®^ HSS C18 SB column (2.1 × 100 mm, 1.8 μm) with an in-line filter and separated by a gradient elution of water containing 0.02% formic acid against an increasing percentage of a mixture of acetonitrile and methanol (volume ratio: 50:50).

For the SA/JA analysis, the compounds were eluted from the column with a mixture of solvents comprised of 1% formic acid in HPLC-grade water (mobile phase A) and 1% formic acid in HPLC-grade methanol (mobile phase B), using a gradient mode. Analytical procedures analogous to those reported by Ross et al. (2004) were employed to determine the quantities of phytohormones in the plant extracts. In brief, the analysis utilizes the multiple reaction monitoring (MRM) function of the MassLynx v4.1 (Waters Inc.) control software. The resulting chromatographic traces are quantified offline by the QuanLynx v4.1 software (Waters Inc.) wherein each trace is integrated and the resulting ratio of signals (non-deuterated/internal standard) is compared with a previously constructed calibration curve to yield the amount of analyte present (ng per sample). Calibration curves were generated from the MRM signals obtained from standard solutions based on the ratio of the chromatographic peak area for each analyte to that of the corresponding internal standard, as described by Ross et al. (2004). The QC samples, internal standard blanks, and solvent blanks were also prepared and analyzed along with each batch of tissue samples. The results were expressed in ng g^−1^ dry weight of the sample for auxin, cytokinin, gibberellin, and ABA, while the results were expressed in ng g^−1^ fresh weight of the sample for SA and JA, both free and conjugated forms. The phytohormones quantified are listed in Supplementary Table 1 for reference.

### 2.4. PEG 8000 plate preparation and experimental setup

Seeds of *Arabidopsis thaliana* were surface-sterilized in 70% alcohol for 2 min and rinsed several times with sterile water. The seeds were sown into petri dishes, on a solid medium containing half strength Murashige and Skoog basal medium (1/2 MS) (Sigma-Aldrich Co, MO, USA, Cat.no. M5519-10L) (Murashige and Skoog, 1962), solidified with 0.8% agar (Fisher Scientific NJ, USA, Cat. no. BP1423-500), and overlaid with different concentrations of PEG 8000 (Sigma-Aldrich Co, MO, USA, Cat. no. 89510-1KG-F). PEG 8000 plate preparations were modeled after the methods outlined by van der Weele et al. (2000) and Verslues and Bray (2004). Three different concentrations of PEG plates, namely, 250, 400, and 550 g L^−1^ PEG 8000, were prepared along with two different concentrations of LCOA and LCOB (10^−6^ and 10^−8^ M) and THA and THB (10^−9^ and 10^−11^ M). The controls consisted of plates with 1/2 MS and agar and another set comprising each of the 250, 400, and 550 g L^−1^ PEG 8000 concentrations. The experiment comprised four replicates per treatment each containing at least 25 seeds, and the experiment was repeated at least twice. The plates were transferred to 4°C in the dark for 48 h (stratification) and then transferred to a growth chamber set at a 16/8 photoperiod, 100 μmole m^−2^ s^−1^ light intensity, and 60–70% relative humidity for drought stress. Seedlings emerging under stress conditions were assessed for fresh weight (FW) and dry weight (DW).

### 2.5. Total proline determination

*Arabidopsis* seedlings were grown on peat pellets, and 3-week-old plants were treated with the abovementioned concentrations of LCO and Th17. Twenty-four hours after the application of the bacterial signal compound treatments, the plants were drought-stressed by withholding water, and plants were sampled on 2 and 4 days of drought stress. These samples were subjected to total proline quantification (Bates et al., 1973). In brief, leaf samples were ground in 3% aqueous sulphosalicylic acid and centrifuged at 10,000 × *g* for 10 min. To 100 μl supernatant, a mixture of 250 μl acid ninhydrin and 250 μl glacial acetic acid was added and mixed well. The reaction mixture was incubated in boiling water for 1 h and the reaction was terminated on ice. To the incubated mixture, 1 ml of toluene was added and shaken vigorously after which the toluene layer was aspirated, and the red color of the toluene layer read against a toluene blank at OD_520_ nm. The amount of proline was calculated as μg proline g^−1^ FW of tissue.

For both elemental analysis and untargeted proteomics, control, and treated plants under 4 days of stress were sampled and processed as described below.

### 2.6. Elemental analysis of the drought-stressed rosettes

The harvested rosettes were dried in an oven at 60°C for 2 days and finely powdered using a coffee grinder. Approximately 2–4 mg of sample was weighed in a microbalance (Sartorius Pro11, Sartorius Corporation, NY, USA), wrapped in a tin capsule (D1008, Isomass Scientific Inc., Calgary, Canada), and subjected to elemental analysis using a ThermoQuest Elemental Analyzer (Model no. NC 2500, Thermo Quest CE Instruments from Isomass Scientific Inc., Calgary, Canada) to determine any shifts in the pattern for percentage nitrogen, carbon, and nitrogen-to-carbon ratio at the end of the experiment at 4 days of drought stress. In brief, the weighed sample in a tin capsule was placed in the autosampler drum, heated to 1,000°C, with a constant flow of helium (carrier gas) enriched with a measured amount of high-purity oxygen to achieve a strongly oxidizing environment. The combustion gas mixture was driven through an oxidation catalyst (Cr_2_O_3_) zone to achieve quantitative oxidation and subsequently through a zone of copper to reduce nitrogen oxides formed during combustion and catalyst oxidation to elemental nitrogen and to scrub excess oxygen. The gas mixture (N, CO_2_, and H_2_O) was passed through a trap containing anhydrone to adsorb water. The resulting components of the combustion mixture were eluted and separated by a Porapak PQS column and subsequently detected by a TCD in the sequence N and CO_2_ (Pella and Colombo, 1973).

### 2.7. Untargeted proteomics of *Arabidopsis* rosettes

#### 2.7.1. Proteomic analysis using LC-MS

*Arabidopsis thaliana plants* were treated with the most effective concentrations of LCOA (10^−6^ M) and THA (10^−9^ M) and water only as a control. After 24 h of treatment, all the plants were subjected to water stress by water withholding. These treatments after water withhold stress will be referred to as drought control (DCtrl), drought LCOA (DLCOA), and drought THA (DTHA) for the proteomics study. Four-day-stressed rosette samples were flash-frozen in liquid nitrogen and stored at −80°C until protein extraction. The total proteins from the samples were extracted using a protein extraction kit (Sigma-Aldrich, PE-2305, St. Louis, MO, USA).

#### 2.7.2. Protein extraction

In brief, the sampled (pool of nine plants per biological replicate) plants were ground to a fine powder in liquid nitrogen. Approximately 100 mg of the fine powder was placed in sterile Eppendorf tubes, and 1 ml of ice-cold methanol (Cat no. 15468-7, Sigma-Aldrich Co., St. Louis, MO, USA) was added. The mix was vortexed, incubated at −20°C for 20 min, and centrifuged (Micro12, Fisher Scientific, Denver Instrument Co., USA) at 13,000 *g* for 30 min at 4°C. The supernatant was discarded, and the procedure was repeated twice, followed by similar incubation and centrifugation in acetone (Cat. no. 179124, Sigma-Aldrich, Co., St. Louis, MO, USA) in order to remove phenolics and secondary metabolites that might otherwise interfere with LC-MS/MS analysis. The RW2 solution was added to the samples after removing acetone, vortexed for 30 s, and incubated at room temperature (22°C) for 15 min. The samples were centrifuged at 13,000 *g* for 30 min, and the supernatant was carefully collected in fresh sterile tubes. The supernatant constituted the total proteins from that sample. Appropriate dilutions of the proteins were quantified using the Lowry method, and samples of 10 μg in 20 μl of 1 M urea were analyzed in Institut de recherches cliniques de Montréal (IRCM) for label-free proteomics using LC-MS/MS.

#### 2.7.3. Protein profiling

The total protein extracts were digested with trypsin and subjected to LC-MS/MS using LTQ Velos Orbitrap (Thermo Fisher, MA, USA). The extracted tandem mass spectra samples were analyzed using Mascot software (Matrix Science, London, UK; version 2.3.02). Mascot was used to search the *Arabidopsis thaliana* database assuming the digestion enzyme trypsin. Mascot was searched with a fragment ion mass tolerance of 0.60 Da and a parent ion tolerance of 15 ppm. Carbamidomethyl of cysteine was specified in Mascot as a fixed modification. Oxidation of methionine was specified in Mascot as a variable modification.

#### 2.7.4. Criteria for protein identification

Scaffold (version Scaffold 4, Proteome Software Inc., Portland, OR) was used to validate MS/MS-based peptide and protein identifications. Peptide identifications were accepted if they could be established at >95.0% probability, as specified by the PeptideProphet algorithm (Keller et al., 2002). Protein identifications were accepted if they can be established at >99.0% probability and contained at least two identified peptides. Protein probabilities were assigned by the ProteinProphet algorithm (Nesvizhskii et al., 2003). Proteins that contain similar peptides and cannot be differentiated based on MS/MS analysis alone were grouped to satisfy the principles of parsimony.

### 2.8. Data analysis

Experiments were conducted following a completely randomized design with sufficient experimental replicates. The SAS statistical package 9.4 (SAS Institute Inc., Cary, NC, USA.) was used for one-way ANOVA, and Tukey's multiple means comparison was used when there was significance at the 95% confidence level, for hormone, plate screening, proline estimation, and elemental analysis data. Data transformation was applied when necessary to meet the criteria for analysis of variance. Data obtained for untargeted proteomics were analyzed using embedded statistical programs in Scaffold 4.4.8. After normalization of the quantitative spectra, the data were subjected to false discovery rate (FDR), fold change between samples, and Fisher's exact test performed with the Benjamini-Hochberg procedure. The FASTA file generated was analyzed using OmicsBox, for the functional annotation and analysis of the protein sequences (Götz et al., 2008, 2011). Apart from these, Enzyme code (EC), KEGG maps, and InterPro motifs were queried directly using the InterProScan web service. The mass spectrometry proteomics data have been deposited to **Mass** Spectrometry **I**nteractive **V**irtual **E**nvironment (MassIVE), with the dataset identifier *PXD040670* and doi: 10.25345/C5H98ZP6R

## 3. Results

### 3.1. Phytohormone quantification

Since previous studies have indicated increased plant growth upon exposure to LCO and Th17, the role of these compounds in triggering hormonal responses during plant growth and regulation was evaluated for the phytohormone categories auxins, cytokinins, gibberellins, abscisic acid, salicylic acid, and jasmonic acid (Supplementary Table 1, for the list of phytohormones tested with abbreviations).

Among the auxins, three auxin conjugates—IAA-Glu (N-(Indole-3-yl-acetyl)-glutamic acid), IAA-Ala (N-(Indole-3-yl-acetyl)-alanine), and IAA-Asp (N-(Indole-3-yl-acetyl)-aspartic acid), were identified. IAA-Glu was present in all samples with a 9.38% increase in LCOA-treated and a 135.98 % increase observed in THA-treated rosettes. IAA-Ala and IAA-Asp were not identified in LCOA-treated samples, while a 112.95% increase in IAA-Ala and a 16.32% increase in IAA-Asp were seen in THA-treated rosettes. However, based on the percentage increase or decrease with reference to control plants, overall IAA levels decreased by approximately 19.94% in LCOA-treated rosettes, while it increased by 6.11% in THA-treated rosettes. The cytokinins *cis*-zeatin-O-glucoside (ZOG), *trans*-zeatin riboside (ZR), and isopentenyladenine (iPA) were observed in all the samples. *cis*-ZR was higher in LCOA by 68.15% and by 48.44% in THA-treated rosettes. iPA was also higher in THA-treated rosettes by 2.98%. The total cytokinin levels, however, were lower in LCOA-treated rosettes by 21.40% and in THA-treated rosettes by 2.08%. Gibberellins were mainly represented by GA19, GA24, GA34, and GA53 in all the samples. While GA19, GA34, and GA53 levels were lower than the control, there was a 7.23% increase in GA24 in LCOA-treated rosettes and a 25.86% increase in THA-treated rosettes. The overall GA content in LCOA and THA rosettes was lower by 16.79 and 16.13%, respectively.

The lyophilized samples contained ABA and related metabolites in substantial amounts for all samples. The amount of ABA and the catabolites were consistently higher in LCOA-treated rosettes. Neo-PA and t-ABA were not identified in LCOA-treated rosettes. Neo-PA was observed in control treatments, and t-ABA was exclusive to THA-treated rosettes. There was a 10.54% increase in overall ABA in LCOA-treated rosettes, while a decrease of 13.47% ABA was observed in THA-treated rosettes. *cis-* and *trans*-abscisic acid (c/t ABA) increased by 15.92% in LCOA and 14.73% in THA treatments. Dihydrophaseic acid (DPA) was 9.69%, and phaseic acid (PA) was 16.31% higher in LCOA than control. Secondary catabolism, such as conjugation resulting in ABA glucose ester (ABAGE), was also detected but was lower in both LCOA and THA as compared to control. Traces of neo-PA were also observed, which indicated that 9′-hydroxylation was also occurring (Supplementary Figure 1 represents the probable pathway for ABA catabolism).

Following LCOA and THA treatments, the amount of free SA increased by 139.59% in LCOA and 85.45% in THA-treated rosettes. Conjugated SA also increased in the treatments by 95.34% in LCOA and 74.28% in THA, respectively. The amount of free JA, however, increased by 24.54% in LCOA but decreased by 13.74% in THA-treated rosettes. However, conjugated JA increased by 30.19 and 13.20% in LCOA and THA, respectively. The quantification data, where the respective phytohormones detected in all the samples, are mentioned in Table 1 with statistical analysis. Supplementary Table 2 provides the percentage distribution of the hormone conjugates and catabolites within the total quantification of each hormone.

**Table 1:** Phytohormones detected using UPLC/LC-MS between control, LCO, and thuricin 17 treatments of 3-week-old *Arabidopsis thaliana* rosettes at 24 h post-treatment.

**Hormone detected**	***p*-value**	**Control**	**LCO**	**Th17**
		**ng/g dry weight**		
Auxin (total)	0.7116	67.59 ± 15.39^a^	54.11 ± 13.95^a^ (−19.94 ↓)	71.72 ± 17.16^a^ (6.11 ↑)
Cytokinin (total)	0.2278	120.55 ± 8.55^a^	94.75 ± 7.97^a^ (−21.40 ↓)	118.04 ± 15.01^a^ (−2.08 ↓)
t-ZR (*trans*-zeatin riboside)	0.0693	39.12 ± 6.48^a^	16.96 ± 2.48^b^ (−56.65 ↓)	28.29 ± 7.83^ab^ (−27.68 ↓)
c-ZR (*cis*-zeatin riboside)	0.4693	12.53 ± 3.31^a^	21.07 ± 5.97^a^ (68.15 ↑)	18.60 ± 5.02^a^ (48.44 ↑)
iPA (isopentenyladenosine)	0.2528	38.53 ± 4.70^a^	27.96 ± 2.69^a^ (−27.43 ↓)	39.68 ± 7.20^a^ (2.98 ↑)
Gibberellic acid (total)	0.4856	36.34 ± 4.22^a^	30.24 ± 3.84^a^ (−16.79 ↓)	30.41 ± 3.82^a^ (−16.32 ↓)
GA24	0.8172	13.69 ± 1.53^a^	14.68 ± 4.47^a^ (7.23 ↑)	17.23 ± 5.15^a^ (25.85 ↑)
GA53	0.6796	12.55 ±1.16^a^	10.57 ± 2.78^a^ (−15.77 ↓)	2.71 ± 2.63^a^ (−78.40 ↓)
Abscisic acid (total)	0.8358	943.51 ± 279.30^a^	1,042.96 ± 320.32^a^ (10.54 ↑)	816.37 ± 250.60^a^ (−13.47 ↓)
c/t ABA (*cis*- and *trans*-abscisic acid)	0.3531	103.48 ± 4.84^a^	119.95 ± 6.16^a^ (15.92 ↑)	118.72 ± 16.45^a^ (14.73 ↑)
DPA (dihydrophaseic acid)	0.9247	550.32 ± 174.79^a^	603.67 ± 201.38^a^ (9.69 ↑)	496.87 ± 193.63^a^ (−9.35 ↓)
ABAGE (abscisic acid glucose ester)	0.355	65.08 ± 8.87^a^	56.91 ± 17.74^a^ (−12.55 ↓)	39.78 ± 6.99^a^ (−40.41 ↓)
PA (phaseic acid)	0.6722	216.95 ± 89.78^a^	252.33 ± 92.59^a^ (16.31 ↑)	154.94 ± 33.52^a^ (−28.58 ↓)
		**ng/g fresh weight**		
**Salicylic acid**				
Total free	0.3583	138.76 ± 32.98^a^	332.46 ± 137.26^a^ (139.59 ↑)	253.17 ± 74.06^a^ (85.45 ↑)
Total conjugated (*n* = 3)	0.2676	2,557.15 ± 770.95^a^	4,995.07 ± 1,135.84^a^ (95.34 ↑)	4,456.63 ± 972.76^a^ (74.28 ↑)
**Jasmonic acid**				
Total free	0.7426	66.06 ± 19.65^a^	82.27 ± 31.28^a^ (24.54 ↑)	56.98 ± 15.61^a^ (−13.74 ↓)
Total conjugated (*n* = 3)	0.4415	0.53 ± 0.14^a^	0.69 ± 0.19^a^ (30.19 ↑)	0.60 ± 0.11^a^ (13.20 ↑)

LS means with the same letter are not significantly different (*n* = 5 unless and otherwise indicated). The numbers in the bracket represent the percentage difference between the control and the treatment).

### 3.2. PEG 8000 plate results

**Figure 1:**
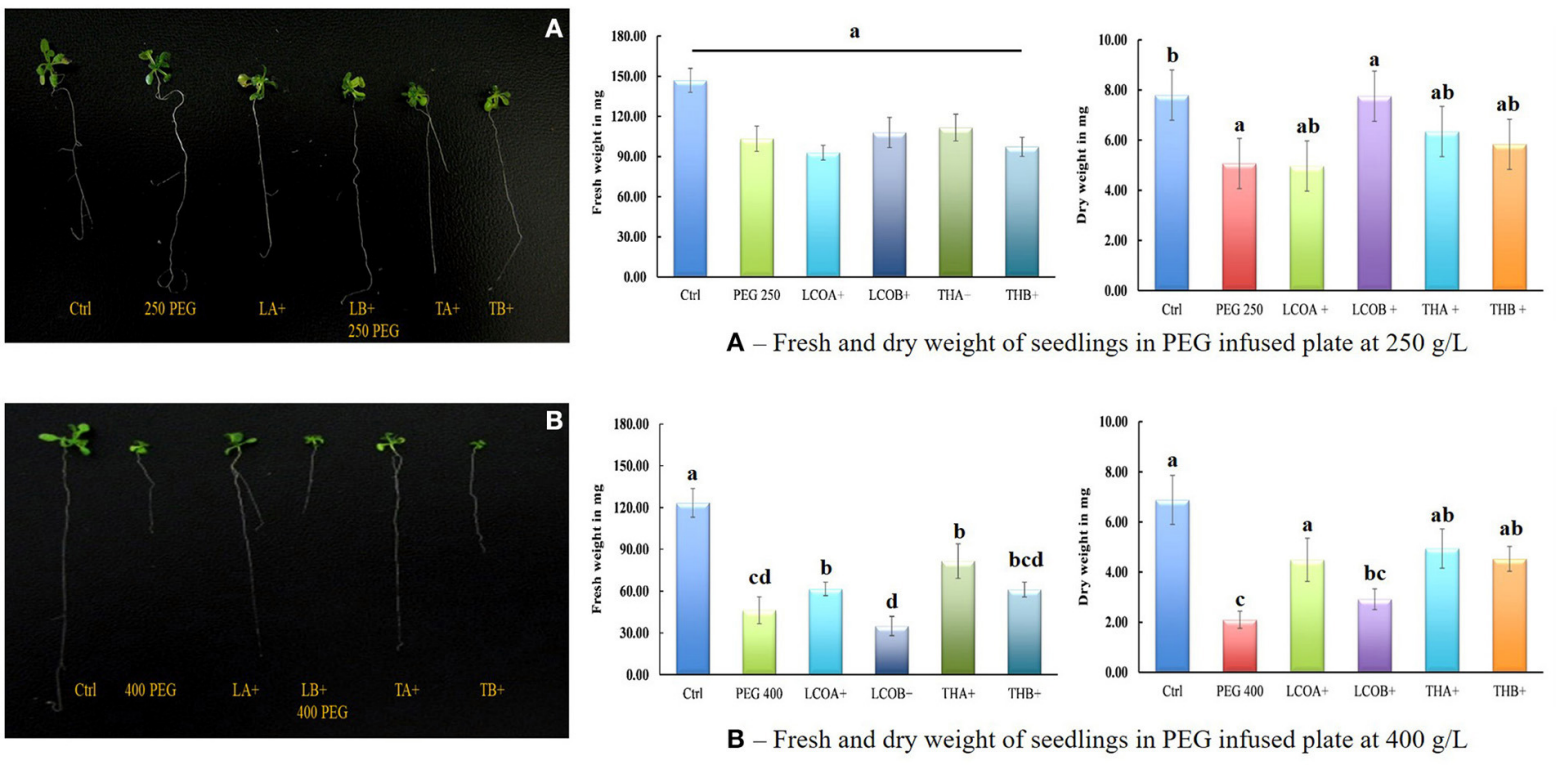
Screening assay in petri-plates for *Arabidopsis thaliana* response to 250 and 400 gL^−1^ PEG 8000 stress in the presence of LCO and Th17, 21 days after imposition of drought stress (control—water; LCOA−10^−6^ M, LCOB−10^−8^ M, THA−10^−9^ M, THB−10^−11^ M; 250 and 400 gL^−1^ PEG 8000 control). **(A)** 250 gL^−1^ PEG 8000 and **(B)** 400 gL^−1^ PEG 8000 stress. Data represents the means and standard error of n = 3, where every n is a pool of technical replicates. Different alphabets on the bar chart represent values determined by Tukey's multiple means comparison to be significantly different at *p* < 0.05 among treatments.

An increase in levels of total ABA in the samples upon exposure to LCOA and THA resulted in the screening of plants at various levels of drought stress using PEG 8000, which is a fast and reliable method followed by most plant biologists studying drought stress research on *Arabidopsis thaliana*. The PEG 250 gL^−1^ plates were intended as a moderate drought stressor for the seedlings. Two levels of LCO (LCOA and LCOB) and Th17 (THA and THB) treatments were studied using this method. An increase in the dry weight of 52.85% was seen in LCOB and 25.05% in THA (*p* = 0.1738). Upon visual comparison, there was little variation in the size of the plants, although Ctrl and Ctrl + PEG did appear to have slightly more vegetative growth, and the root length of the sampled seedlings appeared very uniform except for Ctrl + PEG, which showed longer than average primary root growth (Figure 1A).

With an increase in stress to PEG 400 gL^−1^, which put the *Arabidopsis thaliana* plants under a relatively high level of drought stress, a marked difference in the dry weights of the plants by treatment was observed. Unlike in the PEG 250 gL^−1^ plates, the Ctrl + PEG 400 gL^−1^ treatment showed a dramatic reduction in growth as compared to control plants. Measurements on the fresh (*p* < 0.0001) and dry weights (*p* = 0.0025) of the PEG 400 gL^−1^ plates indicated that LCOA and THA were particularly effective treatments. The size of the plants for Ctrl + PEG, LCOB, and THB was noticeably smaller than the control plant. The plants treated with LCOA and THA were larger and similar in size to the control plants and were able to sustain higher vegetative and root growth than the Ctrl + PEG plants and other treatments (Figure 1B). There was a 113% increase in the dry weight of LCOA and a 132.23% increase in THA-treated plants as compared to PEG 400 gL^−1^ control.

### 3.3. Total proline estimation

**Figure 2:**
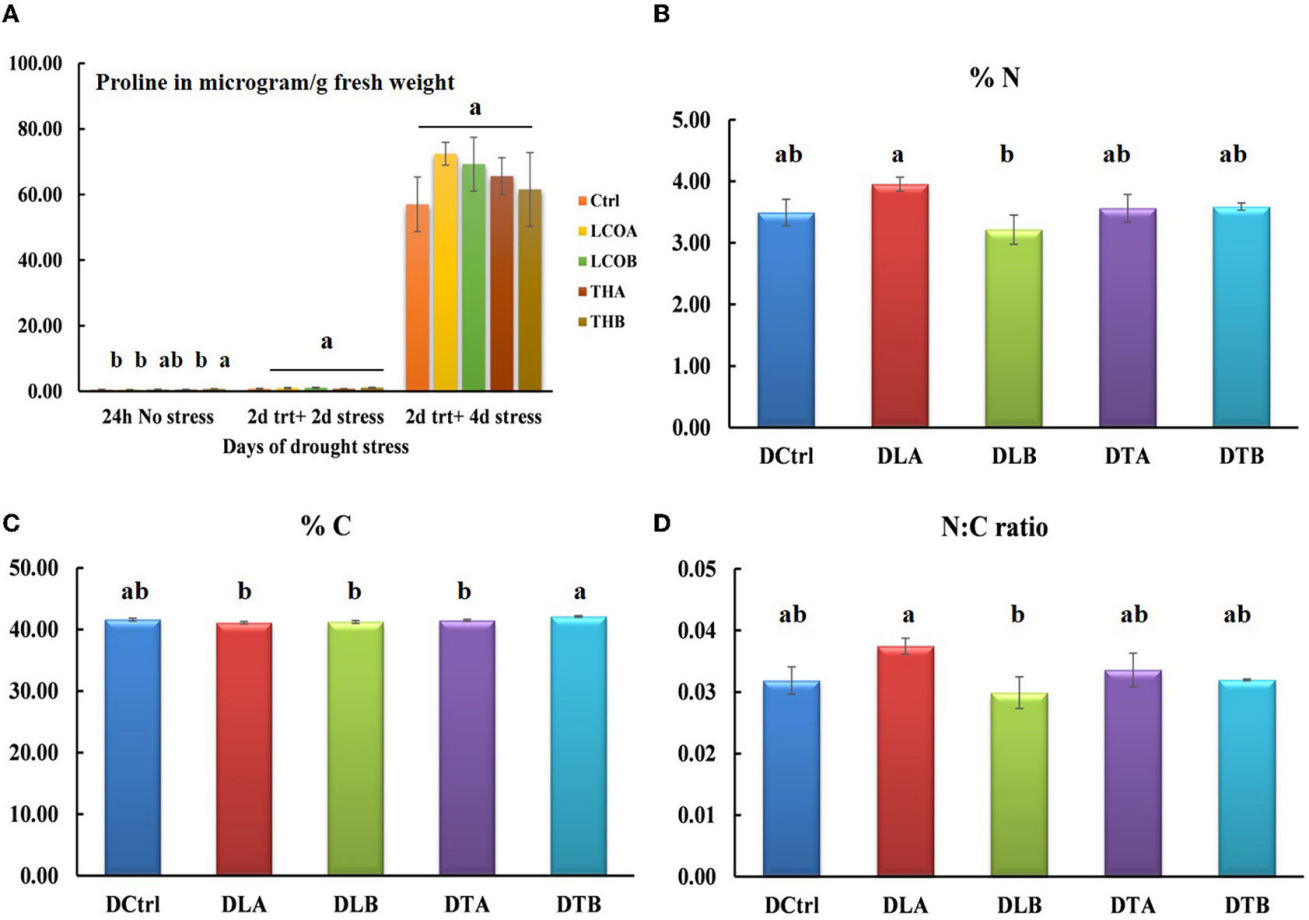
Proline estimation **(A)** and elemental analysis data of *Arabidopsis thaliana* rosettes in response to water withhold stress [**(B)** % nitrogen, **(C)** % carbon, and **(D)** N/C ratio]. Data represents the means and standard error of n = 3, where every n is a pool of technical replicates. Different alphabets on the bar chart represent values determined by Tukey's multiple means comparison to be significantly different at *p* < 0.05 among treatments.

The amounts of ABA and proline have generally been found to co-relate under drought stress, and this study also indicated a moderate increase in free proline in the stress treatments (Figure 2A). At 4 days of drought stress, LCOA (27.01%), LCOB (21.45%), THA (15.05%), and THB (7.96%) had greater levels of proline than the untreated control although not statistically significant at *p* = 0.05. Hence, the 4-day-old rosettes were sampled for elemental analysis and untargeted proteomics in order to capture drought-related stress proteins and to compare with the already published study with the biostimulants and salinity stress.

### 3.4. Elemental analysis

To understand the effect of drought stress on *Arabidopsis thaliana* rosettes' major elements shifts, elemental analysis for nitrogen and carbon percentages in the 2-day treatment + 4 days water withhold was conducted. The rosettes under stress showed statistical significance in the percentage of carbon (*p* = 0.0106), while the percentage of N (*p* = 0.1189) and the N:C ratio (*p* = 0.1315) were not statistically significant, suggesting that carbon metabolism is more affected under drought stress (Figure 2B–% nitrogen, Figure 2C–% carbon, and Figure 2D–N:C ratio; Supplementary Table 3).

To further justify all the above obtained results, untargeted proteome profiling of the rosettes was done using LC-MS/MS.

### 3.5. Label-free proteomics of *Arabidopsis* rosettes

In our previous proteome study, a comparison between *Arabidopsis thaliana* grown under optimal conditions and salt stress was evaluated (Subramanian et al., 2016b). Hence, in this study, only drought-stressed plants were studied, to compare the proteome with the already generated data for optimal and salt stress conditions in response to LCOA and THA treatments. A total of 747 proteins were detected in the rosettes in all the treatments, based on the quantitative value of the identified spectra, and at the 99% protein probability, with two minimum peptides and 95% peptide probability. The proteins detected per biological replicate and associated spectral counts are mentioned in Table 2.

**Table 2:** Total number of proteins identified at 99% protein probability and total spectra at 95% peptide probability with two minimum peptides, representing *n* = 3, where every n is a biological replicate with a pool of nine plants.

**Drought stress**	**DCtrl 1**	**DCtrl 2**	**DCtrl 3**	**DLCOA1**	**DLCOA2**	**DLCOA3**	**DTHA1**	**DTHA2**	**DTHA3**
Proteins	474	487	448	358	550	374	361	534	346
Spectra	5,916	5,424	4,931	4,407	6,946	2,514	4,371	5,446	2,934

In all the contrasts, proteins with known function, hypothetical proteins, putative proteins, and proteins with unknown function were detected based on Fisher's exact test; the fold change of these, in addition to uncharacterized proteins and unnamed protein products, was determined. The proteins found in common in both Fisher's exact test (*p* < 0.05 and below) and fold change (2-fold change and above) among the contrasts are indicated in Table 3. The complete analyzed data are available in Excel sheets for the contrasts as Supplementary Table 4 for Fisher's exact test and Supplementary Table 5 for fold change.

**Table 3:** Proteins found common in both Fisher's exact test and fold change in the contrasts in response to drought stress.

**Identified proteins**	**Accession number**	**MW**	**Fisher's exact test**	**Fold change**	**DCtrl**	**DLCOA**	**Protein function**
PSII 43 KDa protein (chloroplast) (*Arabidopsis thaliana*)	gi|163937814 (+2)	50 kDa	0.0013	3.3	6	20	Photosystem II
Late embryogenesis abundant domain-containing protein (*Arabidopsis thaliana*)	gi|15227965 (+2)	67 kDa	< 0.00010	2.8	19	53	Dehydration protectant
Vegetative storage protein Vsp1 (*Arabidopsis thaliana*)	gi|10129657 (+10)	30 kDa	0.0078	2.2	10	22	Jasmonic acid-related, nitrogen storage
Vegetative storage protein Vsp2 (*Arabidopsis thaliana*)	gi|10129656 (+14)	30 kDa	0.022	2.1	8	17	In response to abscisic acid, jasmonic acid, salt, and water stress
**Identified proteins**	**Accession number**	**MW**	**Fisher's exact test**	**Fold change**	**DCtrl**	**DTHA**	
Protochlorophyllide reductase precursor (*Arabidopsis thaliana*)	gi|14596191 (+19)	43 kDa	0.032	2.8	3	9	Transregulatory protein of thylakoid membrane
Vegetative storage protein Vsp1 (*Arabidopsis thaliana*)	gi|10129657 (+10)	30 kDa	0.017	2.0	8	19	Jasmonic acid-related
**Identified proteins**	**Accession number**	**MW**	**Fisher's exact test**	**Fold change**	**DLCOA**	**DTHA**	
Adenosylhomocysteinase 2 (*Arabidopsis thaliana*)	gi|15229522 (+9)	53 kDa	0.00022	3.9	6	23	Cytokinin and transmethylation
NADP-dependent malic enzyme 2 (*Arabidopsis thaliana*)	gi|15239146 (+4)	64 kDa	0.049	2.4	5	13	Malate metabolism, pentose-phosphate pathway shunt

In Fisher's test, when comparing DCtrl vs. DLCOA, late embryogenesis abundant domain containing PSII 43kDa, vegetative storage protein 1 (Vsp1), pectin acetylesterase, UDP-arabinose mutase, Vsp2, 50S ribosomal, 6-phosphogluconate dehydrogenase, beta-D-glucan exohydrolase-like, and epithiospecifier modifier 1 protein was greater than in the drought control. On the contrary, when comparing DCtrl vs. DTHA, higher levels of glyceraldehyde-3-phosphate dehydrogenase GAPA2, isocitrate dehydrogenase like, pectin acetylesterase, Vsp1, adenosylhomocysteinase 2, protochlorophyllide reductase precursor, non-specific lipid transfer protein, and chloroplastic peroxiredoxin 2E were observed in DTHA. In contrast DLCOA vs. DTHA, DLCOA had higher levels of proteins such as ribulose-1,5-biphosphate carboxylase/oxygenase large subunit, phosphoglycerate kinase-like protein, late embryogenesis abundant, cinnamyl alcohol dehydrogenase 7, glutathione S- transferase, and PSII 43kDa protein, while DTHA had higher levels of adenosylhomocysteinase 2, ferredoxin-dependent glutamate synthase1, mitochondrial chaperonin 10, glyceraldehyde-3-phosphate dehydrogenase GAPA2, 14-3-3-like (general regulatory factor 9), nitrite reductase, RuBisCO small subunit, isocitrate dehydrogenase like, NADP-dependent malic enzyme, and glyceraldehyde-3-phosphate dehydrogenase subunit.

More than 2-fold change proteins were selected for functional description between the contrasts. In DCtrl vs. DLCOA, apart from some of the proteins detected by Fisher's test, proteins such as glycine-rich RNA binding, ribosomal proteins L7Ae/L30e/S12e/Gadd45 family, 60S ribosomal, 26S proteasome, plastid ribosomal, bifunctional and a non-specific lipid transfer, lipase/hydrolase, translocon Tic-40 like, and tryptophan synthase beta chain proteins were increased in DLCOA when compared to DCtrl. A comparison between DCtrl and DTHA found that DTHA exhibited more than 2-fold change levels in the 50S, 60S, and Gadd45 family, L14 ribosomal proteins, and sorbitol dehydrogenase-like proteins than DCtrl. Similarly, a comparison between DLCOA and DTHA showed that DTHA had very specific proteins such as coronatine-induced, ROOTY/SUPERROOT1, 30S, 40S, and 60S ribosomal and chloroplast ribosomal L14, beta-amylase, cysteine synthase, inositol-tetrakisphosphate 1-kinase, geranylgeranyl reductase, allene oxide synthase, PR protein 1, and SOUL heme-binding protein. Proteins for each of the contrasts in both Fisher's test and fold change are mentioned in Table 3 with their functional importance.

Based on OmicsBox results, biological processes, cellular components, molecular function, and the specific enzyme classes affected were identified. In comparison with optimal conditions and 250 mM NaCl salt stress (previously published results in Subramanian et al., 2016b) and the drought control, the functional categories in the treatments all decreased. GO function distribution characteristics of the drought-stressed groups indicated cellular component biogenesis proteins only in the DLCOA treatment. Ribonucleoprotein complex, organelle lumen, non-membrane bounded organelle proteins, and cell periphery proteins were higher in DLCOA than in DTHA. Catabolic process proteins were higher in DLCOA compared to DCtrl and DTHA. Regulation of metabolic process, apoplast proteins, and small molecule binding proteins was higher in DTHA than DLCOA. Graphs for GO functions are shown in Supplementary Figure 2. In the enzyme classes, hydrolases were higher in DLCOA than DCtrl and DTHA. There was not much difference in oxidoreductases between DCtrl and DLCOA, but it was at reduced levels in DTHA. Transferases, lyases, isomerases, and ligases were higher in DTHA than DLCOA, suggesting that LCO and Th17 have different modes of stress alleviation (Table 4).

**Table 4:** Enzyme code distribution drought-stressed groups.

**Main enzyme classes**	**DCtrl**	**DLCOA**	**DTHA**
Oxidoreductases	182	180 (↓ 1.10%)	162 (↓ 10.91%)
Transferases	116	104 (↓ 10.34%)	106 (↓ 8.62%)
Hydrolases	104	106 (↑ 1.92%)	104 (no change)
Lyases	62	54 (↓ 12.9%)	55 (↓ 11.29%)
Isomerases	38	29 (↓ 23.68%)	34 (↓ 10.53%)
Ligases	21	15 (↓ 28.57%)	18 (↓ 14.28%)

The % increase or decrease in the main enzyme classes between treatments in comparison with control drought stress is mentioned in the brackets.

## 4. Discussion

Over the past century, numerous reports have been published on plant hormones playing pivotal roles in diverse plant growth and developmental processes, including responses to abiotic and biotic stresses. Many earlier reports have also shown that higher gene expression need not necessarily match with translation at the systems level. Hence, the direct quantification of available phytohormones, their conjugates, and their catabolites was studied using the UPLC-ESI-MS/MS method. In this study, it was observed that LCO [Nod Bj V(C18:1, MeFuc)] and Th17 provoke different phytohormone responses, 24 h after exposure to the treatments in 3-week-old, root-drenched *Arabidopsis thaliana* rosettes. The role of phytohormones in plant growth and development is a crucial event tightly regulated. A given hormone must be present in the right tissue at the right time to perform the right function. For this to happen, plants have devised a mechanism by which they can store the hormone as free forms or as conjugates or their inactive catabolites which, upon activation, can quickly provide the required level. A study on transcriptional effects of the hormones ABA, GA, auxin, ethylene, cytokinins, brassinosteroids, and jasmonate using microarray data suggests the hormones regulate specific protein families that, in turn, regulate subsequent plant responses (Nemhauser et al., 2006).

In this experiment, THA increased auxin, *cis*-ZR, and iPA components of cytokinins, GA24 of gibberellins, c/t ABA, SA in both free and conjugated forms, and JA in the conjugated form in the rosettes. LCOA treatment increased *cis*-ZR, GA24, total ABA, and ABA components, such as c/t ABA, DPA, and PA, and increased SA and JA in both free and conjugated forms. Auxins function in controlling cell division, lateral root development, gravitropism, and nastic movements and in apical dominance. Although much has been studied about auxins, the mechanisms by which they modulate plant growth response are still elusive. The interaction of auxin and cytokinins is also necessary to control root and shoot apical meristems and flower development (Su et al., 2011; Bielach et al., 2012; Sudre et al., 2013). When no functional role is necessary, the auxin efflux carrier PIN1 is redirected to the vacuoles for degradation, by cytokinin receptors, an effective auxin-cytokinin modulation (Marhavý et al., 2011). Auxin homeostasis is maintained by amino acid conjugates of IAA. While in excess, IAA is reported to conjugate to amino acids such as Ala, Asp, Phe, and Trp (Staswick et al., 2005). The conversion of active IAA to methyl-IAA (MeIAA) is catalyzed by indole-3-acetic acid (IAA)-methyltransferase-1 (IAMT1). IAMT1 plays an important role in leaf development, while MeIAA can also impart a range of responses, such as inducing lateral roots and inhibiting hypocotyl elongation (Li et al., 2008). In this study, most of the IAA conjugation was seen as IAA-Glu, probably suggesting another mechanism of IAA conjugation.

In both LCOA- and THA-treated rosettes, the levels of total GA and cytokinin were lower than the control. GA plays a role in cell wall pectin esterification and microtubule organization (Sánchez-Rodríguez et al., 2010; and references therein). GA24 was the only GA type detected in increased levels in LCOA and THA with reference to control. GA24 is produced by the non-13-hydroxylation pathway of GA metabolism. It is an intermediary type that is converted to GA9 and subsequently to the bioactive GA4 (Li et al., 2016). However, very little is known about the effects of drought stress on GA4 biosynthesis. In plants, isopentenyladenine and its hydroxylated derivative zeatin are the two known active cytokinins (Frébort et al., 2011). A MALDI-TOF/TOF MS analysis of cytokinin signaling indicated early effects of cytokinins in photosynthesis and nitrogen metabolism, light signaling, and the CLAVATA pathway. Major phosphopreoteomic effects were observed in the chloroplast suggesting that cytokinins might play a direct signaling role in chloroplast regulation and functioning (Cerný et al., 2011). LCOA responding with an increase in *cis*-ZR and THA with an increase in *cis*-ZR and iPA suggests a role of active cytokinins in drought stress-related protein regulation. Proteins, including PS II 43 kDa, protochlorophyllide reductase precursor, which is a transmembrane regulatory protein of the thylakoid membrane, and adenosylhomocysteinase 2 that function in transmethylation events, were detected in the protein profiling, suggesting possible roles of these cytokinin components in preventing drought stress-related damage. Cytokinins negatively regulate salt and drought stress, and the balance for this regulation is maintained through ABA metabolism and regulation (Nishiyama et al., 2011). The response of cytokinins in *Arabidopsis thaliana* leaves is necessary to determine the amplitude of the response a plant needs to reciprocate; this is conducted in concert with SA accumulation and defense gene activation in plant–oomycete pathogen interactions such as with *Hyaloperonospora arabidopsidis* isolate Noco2 (Hpa Noco2) (Argueso et al., 2012). It is possible that increases in ABA in LCO-treated rosettes might be involved in the decrease in cytokinin levels. The increase in SA (both free and conjugated forms) in both LCOA and THA might have a significant bearing in modulating drought stress responses.

The signal compound treatments also had an influence on the levels of the phytohormone ABA and ABA catabolite contents. The main ABA metabolism pathway is through 8′-hydroxylation, which results in phaseic acid (PA), a compound that is further reduced to de-oxy phaseic acid (DPA) and modified through secondary catabolism pathways, such as conjugation, resulting in the β-D glucopyranosyl ester of ABA (ABAGE), which is an endogenous conjugate of ABA. Studies in normal bean leaves suggested lower levels of ABA and ABAGE, both of which increased after 24 h of drought stress. However, ABAGE was not the source of induced ABA under water stress, and ABA is reported to be converted to PA or DPA (Neill et al., 2008). Traces of neo-PA were also observed, which indicates the occurrence of 9′-hydroxylation. Neo-PA, as a 9′-hydroxylation product of ABA, is found in several plant tissues and drought-stressed barley and *Brassica napus* seedlings, showing that 9′-hydroxylation is a general pathway for ABA catabolism. In addition, the hydroxylated ABAs have hormonal activity, suggesting that the ABA catabolites play a role in ABA signaling, thus acting as a phytohormone (Zhou et al., 2004). The presence of ABA catabolites suggests that bioactive ABA was probably previously biosynthesized in the tissue and then rapidly metabolized following LCO or Th17 treatments. The presence of Trans-ABA, a product of isomerization of natural ABA under UV light, was observed in plants treated with Th17. It is speculated that one of the ways Th17 modulates UV responses in plants might be by isomerization of natural ABA. In soybean plants treated with Th17, an increase in ABA was observed even 5 days after treatment (Prudent et al., 2014) and this study confirms the role of ABA in *Arabidopsis thaliana*.

The patterns of SA and JA concentration in LCOA- and THA-treated plants suggested an increase in SA for both treatments and a decrease in total JA in THA. Previous studies from our laboratory, on LCO-treated nodulating and non-nodulating soybean cultivars, showed a transient increase in SA at 24 h after treatment (Lindsay, 2007). The results from this study are consistent with this pattern of SA expression. The soybean microarray data also suggested upregulation of stress, SA, and nodulation-related genes. The upregulation of cinnamic acid 4-hydroxylase and not *PAL1* suggests the conversion of cinnamic acid to SA. In addition, the downregulation of isochorismatase hydrolase indicated that isochorismate is available for SA conversion using the isochorismate pathway as well (Lindsay, 2007). JA/SA antagonism is directed at the JA biosynthetic pathway (Leon-Reyes et al., 2010). A 2D-gel, coupled with the MS/MS proteomic approach, to study SA/JA interaction was conducted by dipping 5-week-old *Arabidopsis thaliana* rosettes in SA and JA (1 mM SA, 100 mM MeJA) and harvesting at 24 h after treatment. This experiment suggested that most of the biotic and abiotic stress-related proteins are upregulated by JA, while only a few are induced by SA treatment (Proietti et al., 2013). AhK5 is an *Arabidopsis* histidine kinase that is required by the plant to integrate reactive oxygen species and hormone responses, to impart resistance to biotic stresses, such as *Pseudomonas syringae* pv. *tomato* DC3000 (*Pst*DC3000) and the necrotrophic fungus *Botrytis cinerea*, by controlling SA and JA levels, as a negative regulator in response to salinity stress and for drought stress resistance responses via ABA and NO regulation (Pham and Desikan, 2012). With more information on SA/JA antagonism coming forth, it is not surprising that as SA increased in the LCOA- and THA-treated plants, there was a decrease in free JA in THA treatment.

Since total ABA and catabolites c/t ABA, DPA, and PA were seen to increase in LCOA treatment, and only c/t ABA in THA treatment, there was a need to screen plants for drought stress tolerance using PEG 8000 to validate the effect. The results in this study with PEG plates at a concentration of 250 gL^−1^, a moderate drought stressor, produced little significant results in signal compound-treated plants. In fact, plants treated with 250 gL^−1^ PEG solution alone showed the greatest increase in growth. These plants also showed a visual increase in primary root length, which has previously been shown to be a common result of moderate drought stress (van der Weele et al., 2000). Significant results were seen in PEG plates at a concentration of 400 gL^−1^. Overall, LCOA and THA were by far the most effective treatments for seedling recovery from high drought stress; the treatment concentrations of which have been consistent with all the studies conducted so far.

In *Arabidopsis thaliana*, a natural proline accumulation of 17–26% is seen in flowers and seeds as compared to 1–3% in the rosettes and roots, suggesting that proline is naturally distributed in the plant based on the amount of water in the tissues. Under experimentally induced water stress, an 8- to 10-fold increase in proline was observed under NaCl or KCl stress and a 20-fold increase under PEG-induced drought stress (Chiang and Dandekar, 1995). The presence of proline is significant in the flower-to-seed transition phase and is thought to provide the extra energy required for the reproductive phase (Mattioli et al., 2009). The interaction of mitochondrial protein DFR1 with proline degradation enzymes PDH1/2 and P5CDH has been recently shown to help maintain proline homeostasis during drought stress and recovery (Ren et al., 2018). While most of the stress-related research is conducted with *Arabidopsis thaliana* Col-0, other accessions of *Arabidopsis thaliana* respond to mild drought stress. Drought-sensitive accession ICE163 induced jasmonic acid and anthocyanin pathways for biotic stress defense, while a drought-insensitive accession Yeg-1 had a more classic ABA response for abiotic stress, as seen in Col-0. Most of the accessions tested had similar levels of proline, suggesting that some of the proline responses are baseline responses required by most accessions to maintain homeostasis (Chen et al., 2021). While proline content increased moderately in the LCO and TH17 treatments and was not statistically significantly different, the results conform with the very early findings of Barnett and Naylore (1966) that levels of free proline could increase dramatically, up to 100 times normal, under severe stress conditions, one of the findings that led to using proline quantification to estimate physiological dryness (Bates et al., 1973).

Proteomic studies of drought-stressed *Arabidopsis thaliana* have been reported to result in decreased photosynthesis and that induced a massive proteolysis causing profound changes in cellular proteins and amino acid homeostasis. On average, *Arabidopsis* mesophyll cells contain ~25 billion protein molecules of which 80% of the proteins are localized in the chloroplasts. Drought stress led to the degradation of more than 40% of the leaf proteins, prodding the RuBisCO hexadecamers to double the cellular free amino acids, most of which are channeled to synthesize compatible osmolytes like proline. The remaining proteins are used as an alternate respiratory substrate to compensate for the lack of carbohydrates that are derived from normal photosynthesis (Heinemann et al., 2021). In this study, RuBisCO small subunit proteins and a photosystem II 43 kDa protein were found to increase in LCOA and a protochlorophyllide precursor protein in THA, suggesting that these signal compounds caused a response that protected some of the photosynthesis-associated proteins. An *Arabidopsis* chloroplast lumen-targeted immunophilins (IMMs) with poorly conserved amino acid residues for peptidyl-prolyl isomerase are FKBP16-1, which has been found to be post-transcriptionally regulated under high light and drought conditions. Under light stress, this IMM is found to increase photosynthetic stress tolerance and affects photosystem I-light harvesting complex I and II (PS1-LHC), while drought stress tolerance is increased by the accumulation of Psal protein and regulating its stability (Seok et al., 2014).

Severely drought-stressed *Arabidopsis thaliana* cell suspension callus proteome revealed that overexpression of the ribosome and oxidative phosphorylation-related and endocytosis-related proteins are required for membrane remodeling (Alqurashi et al., 2018). In this study, some of the drought stress-responsive proteins belonged to carbohydrate metabolism pathways such as glyceraldehyde-3-phosphate dehydrogenase, UDP-arabinose mutase, ribulose-1,5-biphosphate carboxylase, sorbitol dehydrogenase, phosphoglycerate kinase-like phosphoenolpyruvate carboxylase, aldehyde dehydrogenase, NADP-dependent malic enzyme, and NADH dehydrogenase flavoprotein 1 that were increased as part of the LCOA and THA responses. Many ribosomal proteins and some oxidative phosphorylation proteins were also specifically increased in these treatments. The result is very similar to the findings in the *Arabidopsis* cell culture proteome under PEG stress that affected the ribosomal binding proteins (RBPs) (Marondedze et al., 2019).

Nitrogen is stored temporarily as vegetative storage proteins in plants, in the form of alkaline phosphatases, chitinases, lectins, and lipoxygenases, and is readily remobilized to buffer plants against abiotic stressors. A mesophyll lipoxygenase ZmLOX6, when overexpressed in corn, showed that the yield of these transgenic hybrids significantly increased under drought stress as compared to their non-transgenic counterparts, by improved ability to store additional nitrogen in the leaves as a vegetative storage protein (Vsp) (Abbaraju et al., 2022). Both LCOA and THA increased Vsp1 and 2 at higher fold and statistical levels, suggesting efficient nitrogen storage under drought stress caused by these signals. Nitric oxide plays a crucial role in the induction of LEA and DREB proteins under drought stress. It is associated with the opening and closing of the stomatal aperture, photosynthesis, proline accumulation (Yu et al., 2014) and signal transduction kinases and phosphatases, metal- and S-nitrosylation, and tyrosine nitration (Grün et al., 2006; Astier and Lindermayr, 2012). *Arabidopsis* cell suspension cultures under PEG-induced drought stress also increased late embryogenesis proteins (LEAs) (Ederli et al., 2019). Both LCOA and THA increase LEA proteins, a nitrite reductase and thioredoxin reductase as specific proteins to drought stress, confirming some of the earlier findings of drought-specific proteome patterns.

Plant thaumatin-like proteins (TLPs) are induced upon osmotic, drought, wounding, and biotic stressors and help by modulating phytohormone signaling pathways and regulating hydrolases, oxidoreductases, and sulfur compound synthesis (Velazhahan et al., 1999; Velazhahan and Muthukrishnan, 2003; Van Loon et al., 2006; Saeidi and Zareie, 2020). TLPs such as bolTLP1 have been found to be expressed under abiotic stress such as in broccoli (He et al., 2021). In this study, LCOA- and THA-treated plants showed higher levels of TLPs than control plants, suggesting that the biostimulants are conferring abiotic stress tolerance and regulating hydrolases, which were seen to increase in LCOA-treated plants.

## 5. Conclusion

Climate change and its effects on crop productivity are a global phenomenon, affecting a wide range of agriculturally intense areas and billions of people. The interactions between atmosphere and climate change drivers have been and will continue to select distinct ecosystem functions, including soil microbial communities. In this study, the biostimulants LCO and Th17 are reported to help alleviate drought stress by regulating drought-specific proteomics responses. LCO at 10^−6^ M and Th17 at 10^−9^ M were the best-suited concentrations, confirming the consistency of the results obtained from our previous research. Many current models predict that one of the effects of global climate change will be an increase in large-scale droughts, and it is possible that signaling compounds, such as LCO and Th17, could play a significant role in mitigating the effects of these droughts on agricultural production.

## Data availability statement

The data presented in the study are deposited in the Mass Spectrometry Interactive Virtual Environment (MassIVE), with the dataset identifier PXD040670.

## Author contributions

SS conceived and designed the experiments and analyzed the data. SS (all the experiments) and EM (petri-plate assay, as a part of under-grad student training) performed the experiments. AS and DS contributed reagents, materials, and analysis tools. SS, EM, and DS wrote the manuscript. All authors contributed to the article and approved the submitted version.

## Supplementary material

Supplementary Figure 1 **(A)** ABA pathway; **(B)** ABA and ABA catabolites in control, LCOA and THA treated plants, 24 h post treatment.

Supplementary Figure 2 Representation of functional classification of GO distribution for biological processes, cellular components, molecular function in drought stressed and signals under water withhold stress treatments in *Arabidopsis thaliana* rosettes.

Supplementary Table 1 Abbreviations of the phytohormones quantified. Phytohormones and their metabolites analyzed in 3-week-old *Arabidopsis thaliana* rosette, 24 h after LCO and Th17 treatments (LCO−10^−6^ M, Th17−10^−9^ M) [UPLC-ESI-MS/MS; Analysis conducted at NRC-PBI Saskatoon, (*n* = 5)].

Supplementary Table 2 Percentage distribution of hormones auxins, cytokinins, gibberellins, and abscisic acid, salicylic acid and jasmonic acid (within the hormone groups) detected using UPLC/LC-MS between control, LCO and Thuricin 17 treatments of 3-week-old *Arabidopsis thaliana* rosettes at 24 h post treatment (*n* = 5 unless otherwise indicated).

Supplementary Table 3 Elemental analysis of 4 days of drought stressed *A. thaliana* rosettes (*n* = 6).

Supplementary Table 4 Fisher's exact test results for *Arabidopsis thaliana* signals group contrasts of signals with drought stress groups.

Supplementary Table 5 Fold-change results for *Arabidopsis thaliana* signals group contrasts of signals with drought stress groups.
