# Supplementary figures and images for: Lipo-chitooligosaccharide and thuricin 17 act as plant growth promoters and alleviate drought stress in Arabidopsis thaliana
Source: Front Microbiol. 2023 Aug 4;14:1184158. doi: 10.3389/fmicb.2023.1184158 (PMC10436337; doi:10.3389/fmicb.2023.1184158)

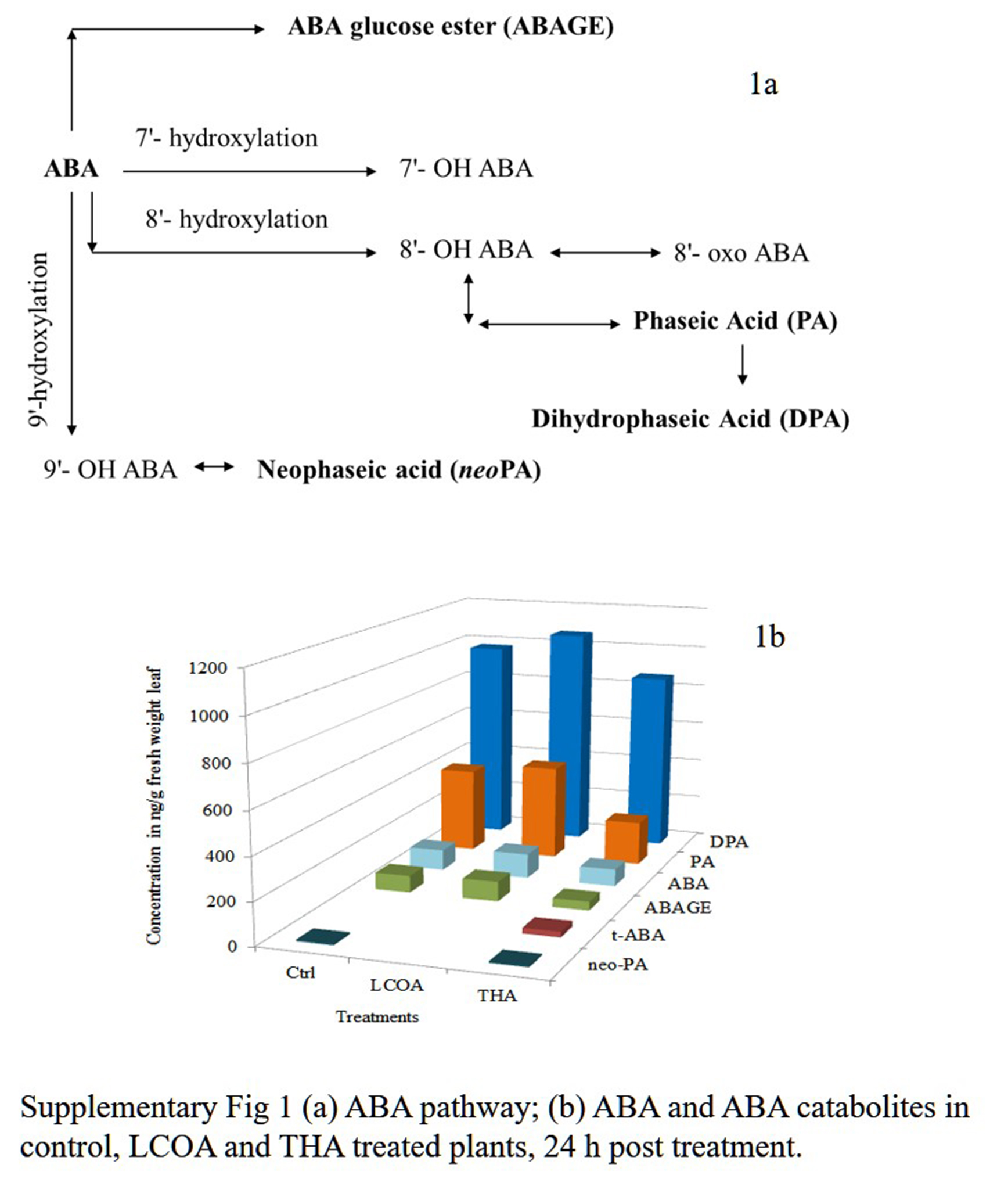

Supplement: Supplementary Figure 1 — (A) ABA pathway; (B) ABA and ABA catabolites in control, LCOA and THA treated plants, 24 h post treatment. [file Image_1.JPEG]

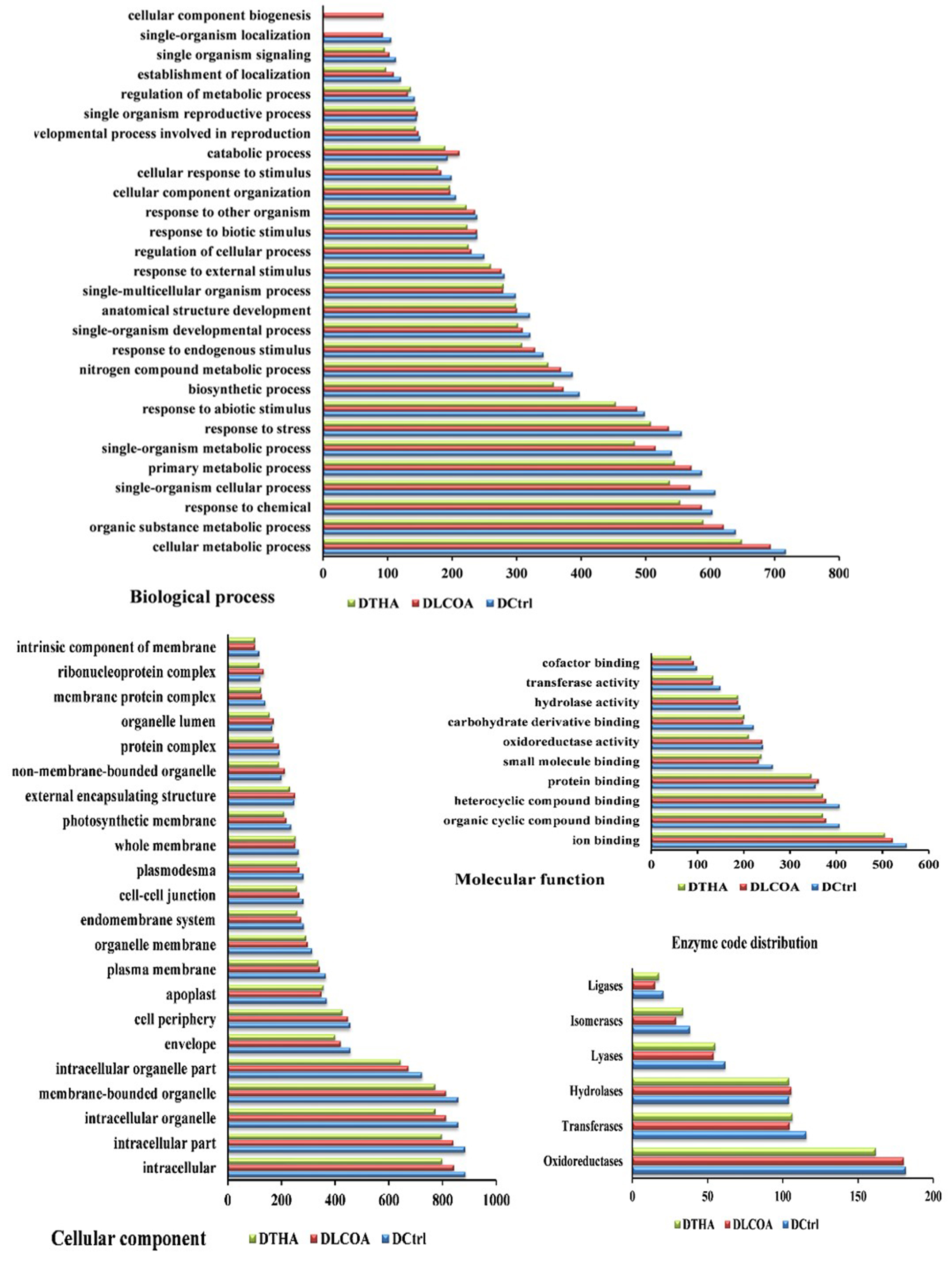

Supplement: Supplementary Figure 2 — Representation of functional classification of GO distribution for biological processes, cellular components, molecular function in drought stressed and signals under water withhold stress treatments in Arabidopsis thaliana rosettes. [file Image_2.JPEG]
