# Supplementary material for: Lipo-chitooligosaccharide and thuricin 17 act as plant growth promoters and alleviate drought stress in Arabidopsis thaliana
Source: Front Microbiol. 2023 Aug 4;14:1184158. doi: 10.3389/fmicb.2023.1184158 (PMC10436337; doi:10.3389/fmicb.2023.1184158)
Supplement: Supplementary Table 1 — Abbreviations of the phytohormones quantified. Phytohormones and their metabolites analyzed in 3-week-old Arabidopsis thaliana rosette, 24 h after LCO and Th17 treatments (LCO−10−6 M, Th17−10−9 M) [UPLC-ESI-MS/MS; Analysis conducted at NRC-PBI Saskatoon, (n = 5)]. [file Table_1.docx]

**Supplementary data**

Supplementary Table 1: Phytohormones and their metabolites analyzed in three-week-old *Arabidopsis thaliana* rosette, 24 h after LCO and Th17 treatments (LCO - 10^-6^ M, Th17 - 10^-9^ M) [UPLC-ESI-MS/MS; Analysis conducted at NRC-PBI Saskatoon, (n = 5)]

| Auxins (IAA) | |
| --- | --- |
| IAA | Indole-3-acetic acid |
| IAA-Asp | N-(Indole-3-yl-acetyl)-aspartic acid |
| IAA-Glu | N-(Indole-3-yl-acetyl)-glutamic acid |
| IAA-Ala | N-(Indole-3-yl-acetyl)-alanine |
| IAA-Leu | N-(Indole-3-yl-acetyl)-leucine |
| IBA | Indole-3-butyric acid |
| Cytokinins (Cyt) | |
| t-ZOG | (trans) Zeatin-O-glucoside |
| c-ZOG | (cis) Zeatin-O-glucoside |
| t-Z | (trans) Zeatin |
| c-Z | (cis) Zeatin |
| dhZ | Dihydrozeatin |
| t-ZR | (trans) Zeatin riboside |
| c-ZR | (cis) Zeatin riboside |
| dhZR | Dihydrozeatin riboside |
| iPA | Isopentenyladenine |
| Gibberellins (GA) | |
| GA1 | Gibberellin 1 |
| GA3 | Gibberellin 3 |
| GA4 | Gibberellin 4 |
| GA7 | Gibberellin 7 |
| GA8 | Gibberellin 8 |
| GA9 | Gibberellin 9 |
| GA19 | Gibberellin 19 |
| GA20 | Gibberellin 20 |
| GA24 | Gibberellin 24 |
| GA29 | Gibberellin 29 |
| GA34 | Gibberellin 34 |
| GA44 | Gibberellin 44 |
| GA51 | Gibberellin 51 |
| GA53 | Gibberellin 53 |
| Abscisic acid (ABA) & metabolites | |
| ABA | cis-Abscisic acid |
| ABAGE | Abscisic acid glucose ester |
| DPA | Dihydrophaseic acid |
| PA | Phaseic acid |
| 7'OH-ABA | 7'-Hydroxy-abscisic acid |
| neo-PA | neo-Phaseic acid |
| t-ABA | trans-Abscisic acid |
| Free and conjugated Salicylic acid (SA) | |
| Free and conjugated Jasmonic acid (JA) | |
