# Supplementary material for: Lipo-chitooligosaccharide and thuricin 17 act as plant growth promoters and alleviate drought stress in Arabidopsis thaliana
Source: Front Microbiol. 2023 Aug 4;14:1184158. doi: 10.3389/fmicb.2023.1184158 (PMC10436337; doi:10.3389/fmicb.2023.1184158)
Supplement: Supplementary Table 2 — Percentage distribution of hormones auxins, cytokinins, gibberellins, and abscisic acid, salicylic acid and jasmonic acid (within the hormone groups) detected using UPLC/LC-MS between control, LCO and Thuricin 17 treatments of 3-week-old Arabidopsis thaliana rosettes at 24 h post treatment (n = 5 unless otherwise indicated). [file Table_2.docx]

**Supplementary data**

Table S2. Percentage distribution of hormones auxins, cytokinins, gibberellins, and abscisic acid, salicylic acid and jasmonic acid (within the hormone groups) detected using UPLC/LC-MS between control, LCO and Thuricin 17 treatments of three-week-old *Arabidopsis thaliana* rosettes at 24 h post treatment (n = 5 unless otherwise indicated).

| Hormone detected | Control | LCO | Thuricin 17 |
| --- | --- | --- | --- |
|  |  |  |  |
| Auxin |  |  |  |
| IAA-Ala (N-(Indole-3-yl-acetyl)-alanine) | 6.64 | 18.01 | 21.00 |
| IAA-Asp (N-(Indole-3-yl-acetyl)-aspartic acid) | nd | nd | 22.97 |
| IAA-Glu (N-(Indole-3-yl-acetyl)-glutamic acid) | 14.14 | 20.95 | 49.54 |
|  |  |  |  |
| Cytokinin | | | |
| t-ZOG (trans) Zeatin-O-glucoside | 29.05 | 30.33 | 29.49 |
| c-ZOG (cis) Zeatin-O-glucoside | 17.47 | 29.88 | 20.62 |
| t-Z (trans-Zeatin) | 0.97 | 0.00 | 1.20 |
| t-ZR (trans-Zeatin riboside) | 32.45 | 17.90 | 23.96 |
| c-ZR (cis-Zeatin riboside) | 10.40 | 22.24 | 15.76 |
| dhZR (Dihydrozeatin riboside) | 1.45 | 1.03 | 1.20 |
| iPA (Isopentenyladenosine) | 31.96 | 29.51 | 33.62 |
|  |  |  |  |
| Gibberellic acid | | | |
| GA19 | 15.80 | 13.44 | 3.41 |
| GA24 | 37.68 | 48.55 | 56.67 |
| GA34 | 11.99 | 3.07 | 8.01 |
| GA53 | 34.54 | 34.94 | 31.92 |
|  |  |  |  |
| Abscisic acid |  |  |  |
| c/t ABA (cis and trans Abscisic acid) | 10.97 | 11.50 | 14.54 |
| DPA (Dihydrophaseic acid) | 58.33 | 57.88 | 60.86 |
| ABAGE (Abscisic acid glucose ester) | 6.90 | 5.46 | 4.87 |
| PA (Phaseic acid) | 22.99 | 24.19 | 18.98 |
| neo-PA (neo-Phaseic acid) | 0.81 | 0.97 | 0.74 |
|  |  |  |  |
| Salicylic acid |  |  |  |
| Total free | 5.15 | 6.24 | 5.38 |
| Total conjugated (n = 3) | 94.85 | 93.76 | 94.62 |
|  |  |  |  |
| Jasmonic acid |  |  |  |
| Total free | 99.21 | 99.16 | 98.95 |
| Total conjugated (n = 3) | 0.79 | 0.84 | 1.05 |
