# Supplementary material for: Lipo-chitooligosaccharide and thuricin 17 act as plant growth promoters and alleviate drought stress in Arabidopsis thaliana
Source: Front Microbiol. 2023 Aug 4;14:1184158. doi: 10.3389/fmicb.2023.1184158 (PMC10436337; doi:10.3389/fmicb.2023.1184158)
Supplement: Supplementary Table 3 — Elemental analysis of 4 days of drought stressed A. thaliana rosettes (n = 6). [file Table_3.docx]

**Supplementary data**

Supplementary Table 3: Elemental analysis of 4 days of drought stressed *A. thaliana* rosettes (n = 6)

| Treatments | % N | ± SE | % C | ± SE | N:C ratio | ± SE |
| --- | --- | --- | --- | --- | --- | --- |
| *p* - value | .1189 | | .0106 | | .1351 | |
| DCtrl | 3.493^ab^ | 0.217 | 41.607^ab^ | 0.241 | 0.032^ab^ | 0.002 |
| DLA | 3.955^a^ | 0.115 | 41.082^b^ | 0.189 | 0.037^a^ | 0.001 |
| DLB | 3.216^b^ | 0.237 | 41.216^b^ | 0.232 | 0.030^b^ | 0.003 |
| DTA | 3.562^ab^ | 0.227 | 41.481^b^ | 0.147 | 0.034^ab^ | 0.003 |
| DTB | 3.590^ab^ | 0.059 | 42.094^a^ | 0.136 | 0.032^ab^ | 0.000 |
